# An in-depth exploration of the association between olanzapine, quetiapine and acute pancreatitis based on real-world datasets and network toxicology analysis

**DOI:** 10.3389/fphar.2025.1529416

**Published:** 2025-05-02

**Authors:** Shuang Wang, Liying Song, Yuanjie Sun, Haonan Zhou, Jia Yao

**Affiliations:** ^1^ Department of Gastroenterology, Third Hospital of Shanxi Medical University, Shanxi Bethune Hospital, Shanxi Academy of Medical Sciences, Tongji Shanxi Hospital, Taiyuan, China; ^2^ Department of Thyroid Surgery, First Hospital of Shanxi Medical University, Taiyuan, China; ^3^ Department of Biliary-Pancreatic Surgery, First Hospital of Shanxi Medical University, Taiyuan, China; ^4^ Department of Vascular Surgery, Third Hospital of Shanxi Medical University, Shanxi Bethune Hospital, Shanxi Academy of Medical Sciences and Tongji Shanxi Hospital, Tongji Medical College of HUST, Taiyuan, China

**Keywords:** acute pancreatitis, antipsychotics, pharmacovigilance, network toxicology, FDA adverse event reporting system

## Abstract

**Objective:**

To combine pharmacovigilance and network toxicology methods to observe the acute pancreatitis (AP) following the use of antipsychotics, and potential toxic mechanisms, and to provide a reference for the safe use of drugs.

**Methods:**

This study combined pharmacovigilance methods using real-world data and network toxicology methods to investigate AP associated with antipsychotics and the potential toxicological mechanism involved. First, the reports of antipsychotics were extracted from the US FDA Adverse Event Reporting System (FAERS), and the signals of AP were detected by four pharmacovigilance algorithms. The gene targets of drugs were predicted using multiple databases. The disease targets of AP were determined by bioinformatics methods. Protein-protein interaction (PPI) analysis was conducted using STRING database, gene ontology (GO) and Kyoto encyclopedia of genes and genomes (KEGG) analysis were also performed through R software. Molecular docking was applied to test the molecular affinity using AutoDock.

**Results:**

The signal intensity of AP was statistically significant in olanzapine, quetiapine, and fluphenazine. Due to the small number of reports associated with AP AEs on fluphenazine, our subsequent studies mainly focused on olanzapine and quetiapine. The results of stratification analysis suggested robustness of our results. Age ≤65, female, and weight >80 kg were identified as risk factors of the development of AP in patients receiving olanzapine, while weight >80 kg and age ≤65 were risk factors of that in patients receiving quetiapine. Network toxicology analysis and molecular docking suggested that olanzapine and quetiapine may exert their toxic effects through acting on hub genes.

**Conclusion:**

The pharmacovigilance analysis investigated the signal intensity, clinical features, risk factors, and onset time of AP associated with olanzapine and quetiapine. Network toxicology analysis suggested that the toxic effects of olanzapine and quetiapine may be related to their hub genes.

## 1 Introduction

Acute pancreatitis (AP) is a complex inflammatory disease caused by acinar cells damage and can cause severe abdominal pain, pancreatic necrosis, and even persistent organ failure (Boxhoorn et al., 2020; Szatmary et al., 2022). It is the most common gastrointestinal disease requiring hospitalization (Boxhoorn et al., 2020). According to previous reports, the global incidence of AP is 34 cases per 100,000 people per year, and it is still rising (Petrov and Yadav, 2019). The severity of AP could be divided into three subtypes: mild, moderate, and severe (Banks et al., 2013). Up to 20%–30% of patients develop moderate or severe AP, which leads to necrosis of the pancreatic or organ failure, and has a mortality of 20%–40% (Boxhoorn et al., 2020). A variety of different factors are thought to be associated with AP, such as alcohol, gallstone, genetic, drugs, infections and so on (Lee and Papachristou, 2019). Drugs accounts for approximately 2.8%–5.3% of all AP cases and are increasingly being recognized as a significant cause of AP (Chadalavada et al., 2020; Vinklerová et al., 2010; Weissman et al., 2020). Recognizing potential drugs which may cause AP is essential for timely discontinuation and avoiding ongoing pancreatic injury (Balani and Grendell, 2008; Naranjo et al., 1981).

Antipsychotics are widely used to treat various psychotic disorders, bipolar disorder, schizophrenia, and adjunctive medication for depressive are their most common indications (Sfera et al., 2024). Since the beginning of the 21st century, the number of individuals using antipsychotics has rapidly increased (Hálfdánarson et al., 2017). As the number of antipsychotics users has increased, many adverse reactions which were not detected in premarket studies have emerged. Several case reports have documented the occurrence of AP following the use of olanzapine and quetiapine. For example, Stelios et al. found a woman developed AP after taking 2 weeks of olanzapine, and had no further episodes during the 2 years without it (Naxakis et al., 2022). Ahmed Naguy et al. reported a female patient who was diagnosed with bipolar I disorder developed AP after receiving quetiapine. And she was in full remission 6 months after discontinuing quetiapine (Naguy and Elsori, 2018). Although these studies have made reference to the occurrence and clinical features of AP induced by olanzapine and quetiapine, they are insufficient to assess the real situation due to issues such as small sample sizes. Further research is needed to better understand the relationship between these drugs and AP. However, there have been no studies so far that comprehensively analyze the impact of these drugs on AP using large databases and further explore their potential molecular mechanisms.

Pharmacovigilance is a great approach to detect, assess, and understand adverse events induced by drugs using real-world data (Beninger, 2018). The United States (United States) Food and Drug Administration (FDA) adverse event reporting system (FAERS) database recorded a large amount of adverse event reports reported from the real-world, providing great supports to the post-marketing pharmacovigilance study (Duan et al., 2017). Therefore, we conducted this pharmacovigilance study using the FAERS database to investigate the relationship between antipsychotic drugs and AP, including clinical features, onset time, and so on.

Based on a comprehensive understanding of the risk and clinical characteristics of antipsychotic-induced AP, exploring its toxicological mechanisms helps to avoid and accurately deal with antipsychotic-induced AP. Network toxicology methods assist us in quickly and effectively understanding the toxicological mechanisms of drugs. In this study, we integrated pharmacovigilance and network toxicology to initially elucidate the risk characteristics, clinical features, and toxicological mechanisms of antipsychotic-induced AP.

## 2 Materials and methods

### 2.1 Data sources

Based on the Anatomical Therapeutic Chemical (ATC) classification system, we identified 63 kinds of antipsychotic drugs (Supplementary Table S1). Data in this study was extracted from the FAERS database, covering the period from the first quarter (Q1) of 2004 to the second quarter (Q2) of 2024. Following the FDA’s recommendation of eliminating duplicate reports, we retained a single report per patient across different reporting sources (Shu et al., 2022). We classify drugs in FAERS into four categories: primary suspect drug (PS), secondary suspect drug (SS), concomitant (C), and interacting (I) based on the anticipated degree of AEs involvement. This analysis included only reports containing the PS drug code.

### 2.2 Identification of AP report

Adverse drug reactions (ADRs) are identified using standardized medical terminology known as preferred terms (PTs), as coded by the Medical Dictionary for Regulatory Activities (MedDRA). Standardized MedDRA Queries (SMQs) consists of multiple PTs representing related medical conditions. It aids in the retrieval of interesting cases from the MedDRA-coded database and enhances the detection and assessment of ADR signals (Mozzicato, 2007). An SMQ provides two search types to identify target cases, namely, broad-scope search and narrow-scope search (Li et al., 2023). The broad-scope search contains all the PTs potentially related to the condition or area of interest, while the narrow-scope search focuses solely on PTs directly associated with the condition or area (Li et al., 2023). To ensure the specificity of target adverse event (AE) report identification, we applied the narrow-scope search for “Acute pancreatitis (SMQ)” as per MedDRA version 27.0. If an AE report included any PT in Supplementary Table S2, we consider it the target report.

### 2.3 Signal mining

We used the reporting odds ratio (ROR), proportional reporting ratio (PRR), Bayesian confidence propagation neural network (BCPNN), and the multiple Gamma Poisson reduction method (EBGM) algorithms to investigate the signals associated with AP AEs, based on non-proportional analyses and the core principles of Bayesian analysis. Signals satisfying all four algorithms simultaneously were considered positive. The detailed information of the formulas and scoring thresholds for the four algorithms were shown in Table 1.

**TABLE 1:** Formulas and signal detection standards of ROR, PRR, BCPNN, and EBGM.

Algorithm	Equation	Criteria
ROR	$\text{ROR}=\frac{\text{ad}}{\text{bc}}$ ${95\%\;\text{CI}=e}^{\ln\left(ROR\right)\pm 1.96\sqrt{\left(\frac{1}{a}+\frac{1}{b}+\frac{1}{c}+\frac{1}{d}\right)}}$	a≥3, the lower limit of 95%CI > 1
PRR	$\text{PRR}=\frac{a/\left(a+b\right)}{c/\left(c+d\right)}$ $\chi^{2}=\frac{{\left(ad-bc\right)}^{2}\left(a+b+c+d\right)}{\left(a+b\right)\left(c+d\right)\;\left(a+c\right)\left(b+d\right)}$ ${95\%\;\text{CI}=e}^{\ln\left(PRR\right)\pm 1.96\sqrt{\left(\frac{1}{a}-\frac{1}{a+b}+\frac{1}{c}-\frac{1}{c+d}\right)}}$	PRR≥2, χ^2^ ≥ 4, a≥3
BCPNN	$\text{IC}=\log_{2}\frac{p\left(x,y\right)}{p\left(x\right)p\left(y\right)}=\log_{2}\frac{a\left(a+b+c+d\right)}{\left(a+b\right)\left(a+c\right)}$ $E\;\left(\text{IC}\right)=\log2\;\frac{\left(a+\gamma 11\right)\left(a+b+c+d+\alpha \right)\left(a+b+c+d+\beta \right)}{\left(a+b+c+d+\gamma \right)\left(a+b+\alpha 1\right)\left(a+c+\beta 1\right)}$ $V\left(\text{IC}\right)=\frac{1}{{\left(\ln2\right)}^{2}}\left\{\left[\frac{\left(a+b+c+d\right)-a+\gamma -\gamma 11}{\left(a+\gamma 11\right)\left(1+a+b+c+d+\gamma \right)}\right]+\left[\frac{\left(a+b+c+d\right)-\left(a+b\right)+\alpha -\alpha 1}{\left(a+b+\alpha 1\right)\left(1+a+b+c+d+\alpha \right)}\right]+\left[\frac{\left(a+b+c+d\right)-\left(a+c\right)+\beta -\beta 1}{\left(a+c+\beta 1\right)\left(1+a+b+c+d+\beta \right)}\right]\right\}$ $\gamma =\gamma 11\;\frac{\left(a+b+c+d+\alpha \right)\left(a+b+c+d+\beta \right)}{\left(a+b+\alpha 1\right)\left(a+c+\beta 1\right)}$ $\text{IC}-2\text{SD}=E\;\left(\text{IC}\right)-2\;\sqrt{V\left(IV\right)}$ α1=β1=1,α=β=2,γ11=1	a≥3, IC025 > 0
EBMG	$\text{EBGM}=\frac{a\left(a+b+c+d\right)}{\left(a+c\right)\left(a+b\right)}$ $\text{EBGM}05=e^{{\ln\left(EBGM\right)-1.64\left(\frac{1}{a}+\frac{1}{b}+\frac{1}{c}+\frac{1}{d}\right)}^{0.5}}$	a>0, EBGM05 > 2

Equation: a, the number of reports simultaneously containing the target drug and target adverse events; b, the number of reports simultaneously containing the target drug and other adverse events; c, the number of reports simultaneously containing the other drugs and target adverse events; d, number of reports simultaneously containing the other drugs and the other adverse events.

### 2.4 Time‐to‐onset analysis

To describe the latency of AP induced by non-selective RET MKIs, we performed time to onset (TTO) analysis. TTO was determined by calculating the duration between the onset of the AEs (EVENT_DT recorded in the DEMO file) and the initiation of drug treatment (START_DT recorded in the THER file). Only reports with available TTO data were analyzed to ensure the accuracy of the calculations. The median and quartiles were also used to describe TTO data. WSP test, a probability distribution, was performed to describe the varying ratio of the time to onset. The α (scale parameter) and β (shape parameter) were used to describe three types of hazard models as follows: (i) when β > 1 and it’s 95% CI > 1, the risk of AE was considered to have increased over time (wear-out failure type); (ii) when β < 1 and it’s 95% CI < 1, the risk of AE was considered to have decrease over time (early failure type); (iii) when β <= 1 and it’s 95% CI includes the value 1, the risk of AE was considered to have constant or near-constant over time (random failure type).

### 2.5 Model construction and evaluation

Using logistic regression, we investigated the risk factors of the development of AP in patients receiving olanzapine or quetiapine. Additionally, we constructed a nomogram to estimate the probability of developing AP in patients receiving olanzapine or quetiapine. Each variable corresponds to a line segment with scales marked, which represents the range of values that the variable can take, and the length of the line segment reflects the contribution of that factor to the occurrence of AP. To evaluate the predictive performance of the model, we also calculate the area under the curve (AUC) of receiver operating characteristics (ROC).

### 2.6 WGCNA (weighted gene co-expression network analysis)

GSE194331, which contained peripheral blood gene expression data of 87 AP patients and 32 healthy patients, was obtained from the Gene Expression Omnibus (GEO) database (https://www.ncbi.nlm.nih.gov/geo/). WGCNA analysis was applied to obtain hub genes that related to AP. The pickSoftThreshold function in “WGCNA” R package was employed to establish the soft threshold β. Modules (minModuleSize = 60) were identified by hierarchical clustering and similar modules were subsequently merged (abline = 0.25). The module eigengene (ME) and module membership (MM) were used to clarify important modules associated with clinical traits. Finally, the correlation between the modules and the clinical data was calculated to identify significant clinical modules.

### 2.7 Drug targets screening

The 3D structures and SMILES of the olanzapine and quetiapine were both sourced from the PubChem database (https://pubchem.ncbi.nlm.nih.gov/). Utilizing this structural data, potential drug targets were identified through a series of computational analyses across multiple platforms: the Comparative Toxicogenomics Database (https://ctdbase.org/), Swiss Target Prediction (http://www.swisstargetprediction.ch/), BindingDB (http://bindingdb.org/bind/index.jsp), and TargetNet (http://targetnet.scbdd.com/home/index/). The target prediction results underwent a standardization process using the UniProt and STRING databases.

### 2.8 Identification of potential drug targets in AP

Intersecting the genes in significant clinical modules identified by WGCNA with drug targets screened from multiple databases, the potential targets of olanzapine and quetiapine in AP were determined.

### 2.9 Construction of protein-protein interaction (PPI) network

To ascertain information regarding interactions among proteins, data pertaining to potential targets of olanzapine and quetiapine in AP were submitted to the esteemed STRING database (https://string-db.org/) (Szklarczyk et al., 2019). The specified analysis species is “*Homo sapiens*,” with a minimum interaction score threshold of 0.15. The results were obtained in TSV format and imported into Cytoscape 3.10.2 to visualize the interactions of the PPI network. Utilizing the exceptional CytoNCA Cytoscape plugin, we identified pivotal genes in the PPI network. The Degree centrality (DC) was calculated using the CytoNCA plugin. DC represents the number of connections between nodes. The higher the DC value of a node, the stronger the interaction with other nodes and their regulated downstream nodes. In this study, the larger and redder nodes indicated genes with higher DC value.

### 2.10 Functional analyses

The Gene Ontology (GO) and Kyoto Encyclopedia of Genes and Genomes (KEGG) enrichment analysis were also conducted using the “clusterProfiler” R package (Subramanian et al., 2005).

### 2.11 Molecular docking

The mol2 structure files of olanzapine and quetiapine were input into AutoDockTools (v1.5.7), set as ligands, and saved in pdbqt format. The 3D structure files of human target proteins were searched and downloaded from PDB database (https://www1.rcsb.org/). PyMOL 2.3.0 was used to delete the water molecule and the primary ligand of the downloaded target protein.

The target proteins were input in PDB format into AutoDockTools, preprocessed for dehydration and hydrogenation, and output in PDBQT format. The docking box was set to encompass the entire protein structure. Finally, molecular docking validation was performed using AutoDock Vina, outputting the binding results of the protein with olanzapine and quetiapine and visualizing the one with the minimum free energy.

## 3 Results

### 3.1 The signal spectrum of AP in antipsychotics therapy

The ATC code of antipsychotics is N05A, which involves a total of 63 drug active ingredients (Supplementary Table S1). Among them, the signal intensity of AP AEs, which was calculated by four algorithms, ROR, PRR, BCPNN and EBGM, was statistically significant in olanzapine, quetiapine, and fluphenazine (Table 2). Among the three drugs, quetiapine caused the most AP reports (N = 3531) and had the highest signal intensity [ROR (95% CI): 10.58 (10.23–10.95); PRR (95% CI): 10.46 (10.42–10.49), χ^2^: 28,731.72; BCPNN (IC025): 1.65; EBGM05: 9.71; adjust.P value = 0]. Furthermore, Supplementary Table S3 shows the non-significant signals related to AP as associated with antipsychotic drugs.

**TABLE 2:** ROR, PRR, BCPNN, and EBGM for antipsychotics-associated AP with significant signal intensity.

Drug	Number	ROR (95%CI)	PRR (95%CI)	χ^2^	BCPNN (IC025)	EBGM (EBGM05)	adjust.P value
Olanzapine	1,458	6.35 (6.03–6.69)	6.31 (6.26–6.36)	6,390.18	0.97	5.94	0
Quetiapine	3,531	10.58 (10.23–10.95)	10.46 (10.42–10.49)	28,731.72	1.65	9.71	0
Fluphenazine	11	5.69 (3.14–10.3)	5.66 (5.07–6.24)	42.21	0.83	3.44	8.36E-10

### 3.2 Descriptive analysis

**FIGURE 1:**
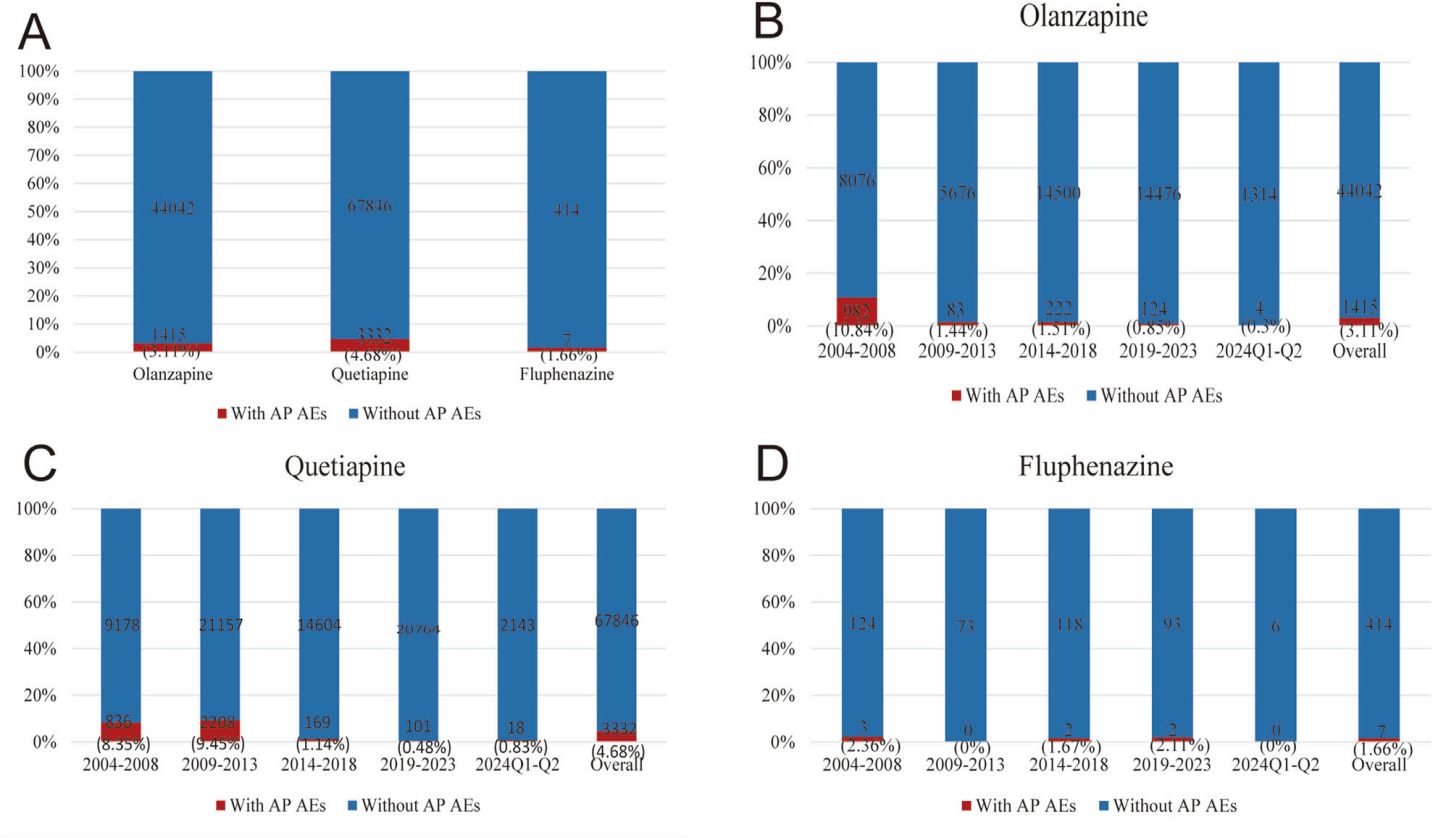
Statistics on the reporting ratio of AP following the use of olanzapine, quetiapine, and fluphenazine reported in FAERS during 2004Q1–2024Q2. **(A)** A comparison of the number of olanzapine, quetiapine, and fluphenazine with AP versus olanzapine, quetiapine, and fluphenazine reports without AP. **(B)** A comparison of the number of olanzapine reports with AP and the number of olanzapine reports without AP in FAERS every 5 years. **(C)** A comparison of the number of quetiapine reports with AP and the number of quetiapine reports without AP in FAERS every 5 years. **(D)** A comparison of the number of fluphenazine reports with AP and the number of fluphenazine reports without AP in FAERS every 5 years.

After observing statistically significant signal intensity of AP in olanzapine, quetiapine, and fluphenazine, we further investigated the clinical features of AP caused by the three drugs. From the first quarter of 2004 to the second quarter of 2024, after excluding duplicate reports, 18,182,912 AE reports were identified in the FAERS database. 45,457 reports identified as related to the use of olanzapine, 71,178 reports identified as related to the use of quetiapine, and 421 reports identified as related to the use of perphenazine. Among them, 1,415 (3.11%) reports on olanzapine, 3,332 (4.68%) reports on quetiapine, and 7 (1.66%) reports on fluphenazine were identified to be associated with AP (Figure 1A). As shown in Figures 1B–D, the number of AP AEs of olanzapine, quetiapine, and fluphenazine every 5 years showed a small proportion of the total case numbers (0.3%–0.84% on olanzapine, 0.48%–9.45% on quetiapine, 0%–2.36% on fluphenazine).

As shown in Table 3, in patients receiving olanzapine, AP AEs were more prevent in males than females (663, 46.9% vs. 586, 41.4%). The highest number of reports came from individuals aged older than or equal to 65 years and younger than 85 years (801, 56.6%). The weight group with the highest number reports was 50∼100 kg (530, 37.5%). The number of reports from the United States was the highest (1,145, 80.90%). In patients receiving quetiapine, AP AEs were more prevent in females than males (1750, 52.5% vs. 1,444, 43.3%). The age group with the highest number of reports was also 18–65 years old (1,042, 31.3%), and the weight group with the highest number of reports was 50∼100 kg (825, 24.8%). The United States of America had the highest number of reports (3082, 92.50%). Interestingly, all the 7 reports on fluphenazine which were associated with AP AEs were identified to be males, and were older than or equal to 18 years and younger than 65 years old. However, due to the limited reports on the association between fluphenazine and AP AEs, our subsequent studies mainly focused on olanzapine and quetiapine.

**TABLE 3:** Clinical characteristics of patients with olanzapine-, quetiapine-, fluphenazine-associated AP.

Characteristics		Olanzapine	Quetiapine	Fluphenazine
Gender	Male	663 (46.9%)	1,444 (43.3%)	7 (100%)
	Female	586 (41.4%)	1750 (52.5%)	0 (0%)
	Unknown	166 (11.7%)	138 (4.1%)	0 (0%)
Age	<18	16 (1.1%)	32 (1.0%)	0 (0%)
	18 ≤ and < 65	801 (56.6%)	1,042 (31.3%)	7 (100%)
	65 ≤ and ≤ 85	42 (3.0%)	32 (1.0%)	0 (0%)
	>85	2 (0.1%)	2 (0.1%)	0 (0%)
	Unknown	554 (39.2%)	2,224 (66.7%)	0 (0%)
Weight	<50 kg	29 (2.0%)	33 (1.0%)	0 (0%)
	50∼100 kg	530 (37.5%)	825 (24.8%)	3 (42.9%)
	>100 kg	190 (13.4%)	394 (11.8%)	0 (0%)
	Unknown	666 (47.1%)	2080 (62.4%)	4 (57.1%)
Reporter	Consumer	411 (29.0%)	80 (2.4%)	0 (0%)
	Health Professional	52 (3.7%)	18 (0.5%)	0 (0%)
	Physician	202 (14.3%)	274 (8.2%)	0 (0%)
	Other health-professional	35 (2.5%)	258 (7.7%)	5 (71.4%)
	Pharmacist	20 (1.4%)	41 (1.2%)	0 (0%)
	Lawer	675 (47.7%)	611 (18.3%)	0 (0%)
	Unknown	20 (1.4%)	2050 (61.5%)	2 (28.6%)
Country	Top five	The United States of America: 1,145 (80.90%)	The United States of America: 3,082 (92.50%)	Croatia: 5 (71.50%)
		Canada: 29 (2.00%)	France: 40 (1.20%)	India: 1 (14.3%)
		The United Kingdom of Great Britain and Northern Ireland: 22 (1.60%)	The United Kingdom of Great Britain and Northern Ireland: 39 (1.20%)	The United States of America: 1 (14.3%)
		India: 16 (1.10%)	Canada: 31 (0.90%)	
		Greece: 9 (0.60%)	Germany: 20 (0.60%)	
Outcome	Death	105 (5.0%)	321 (7.6%)	6 (46.2%)
	Life-Threatening	68 (3.3%)	81 (1.9%)	0 (0%)
	Disability	16 (0.8%)	44 (1.0%)	0 (0%)
	Hospitalization - Initial or Prolonged	708 (34.0%)	740 (17.6%)	4 (30.8%)
	Other Serious (Important Medical Event)	1,175 (56.5%)	2,994 (71.2%)	3 (23.1%)
	Required Intervention to Prevent Permanent Impairment/Damage	7 (0.3%)	17 (0.4%)	0 (0%)
	Nonserious outcome	1 (0.0%)	7 (0.2%)	0 (0%)
Year	2004	41 (2.9%)	5 (0.2%)	0 (0%)
	2005	123 (8.7%)	6 (0.2%)	0 (0%)
	2006	183 (12.9%)	12 (0.4%)	0 (0%)
	2007	626 (44.2%)	743 (22.3%)	3 (42.9%)
	2008	9 (0.6%)	70 (2.1%)	0 (0%)
	2009	28 (2.0%)	1,337 (40.1%)	0 (0%)
	2010	15 (1.1%)	641 (19.2%)	0 (0%)
	2011	30 (2.1%)	34 (1.0%)	0 (0%)
	2012	7 (0.5%)	56 (1.7%)	0 (0%)
	2013	3 (0.2%)	140 (4.2%)	0 (0%)
	2014	5 (0.4%)	30 (0.9%)	0 (0%)
	2015	185 (13.1%)	40 (1.2%)	0 (0%)
	2016	13 (0.9%)	48 (1.4%)	0 (0%)
	2017	7 (0.5%)	10 (0.3%)	0 (0%)
	2018	12 (0.8%)	41 (1.2%)	2 (28.6%)
	2019	15 (1.1%)	28 (0.8%)	2 (28.6%)
	2020	11 (0.8%)	16 (0.5%)	0 (0%)
	2021	48 (3.4%)	26 (0.8%)	0 (0%)
	2022	18 (1.3%)	15 (0.5%)	0 (0%)
	2023	32 (2.3%)	16 (0.5%)	0 (0%)
	2024	4 (0.3%)	18 (0.5%)	0 (0%)

### 3.3 Stratification analysis

We employed three different stratification strategies: gender (male and female), age (<=65 years and >65 years), and weight (<=80 kg and >80 kg) to enhance the robustness of the results. As shown in Table 4, in patients receiving olanzapine or quetiapine, all the four algorithms: ROR, PRR, BCPNN, and EBGM, simultaneously showed positive AP signals in subgroups of gender, weight, and age <=65 years, indicating the associations between AP and olanzapine or quetiapine persisted.

**TABLE 4:** Stratification analysis of olanzapine- and quetiapine-induced AP.

Drug	Subgroup		Number	ROR (95%CI)	PRR (95%CI)	χ^2^	EBGM05	IC025	adjusted.P
Olanzapine	Age	≤65	858	5.23 (4.89–5.6)	5.2 (5.13–5.27)	2,842.69	4.81	0.68	0
		>65	38	1.83 (1.33–2.52)	1.83 (1.51–2.15)	14.3	1.4	−0.8	0.00024
	Gender	Male	693	4.89 (4.54–5.28)	4.86 (4.79–4.94)	2079.73	4.48	0.59	0
		Female	597	7.18 (6.62–7.79)	7.13 (7.05–7.21)	3,094.03	6.56	1.15	0
	Weight	≤80	319	9.19 (8.21–10.27)	9.09 (8.98–9.2)	2,240.14	8.09	1.48	0
		>80	460	8.95 (8.15–9.84)	8.81 (8.72–8.91)	3,063.09	7.85	1.42	0
Quetiapine	Age	≤65	1,184	5.09 (4.81–5.4)	5.06 (5.01–5.12)	3,733.6	4.69	0.63	0
		>65	34	1.1 (0.79–1.55)	1.1 (0.77–1.44)	0.33	0.83	−1.53	0.63
	Gender	Male	1,522	9.76 (9.27–10.28)	9.63 (9.58–9.68)	11,187.77	8.8	1.53	0
		Female	1871	11.65 (11.12–12.21)	11.52 (11.48–11.57)	17,001.98	10.52	1.79	0
	Weight	≤80	529	7.7 (7.06–8.41)	7.64 (7.55–7.72)	2,922.26	6.83	1.21	0
		>80	898	8.53 (7.96–9.14)	8.41 (8.34–8.48)	5,407.9	7.38	1.3	0

### 3.4 Analysis of factors influencing AP

**FIGURE 2:**
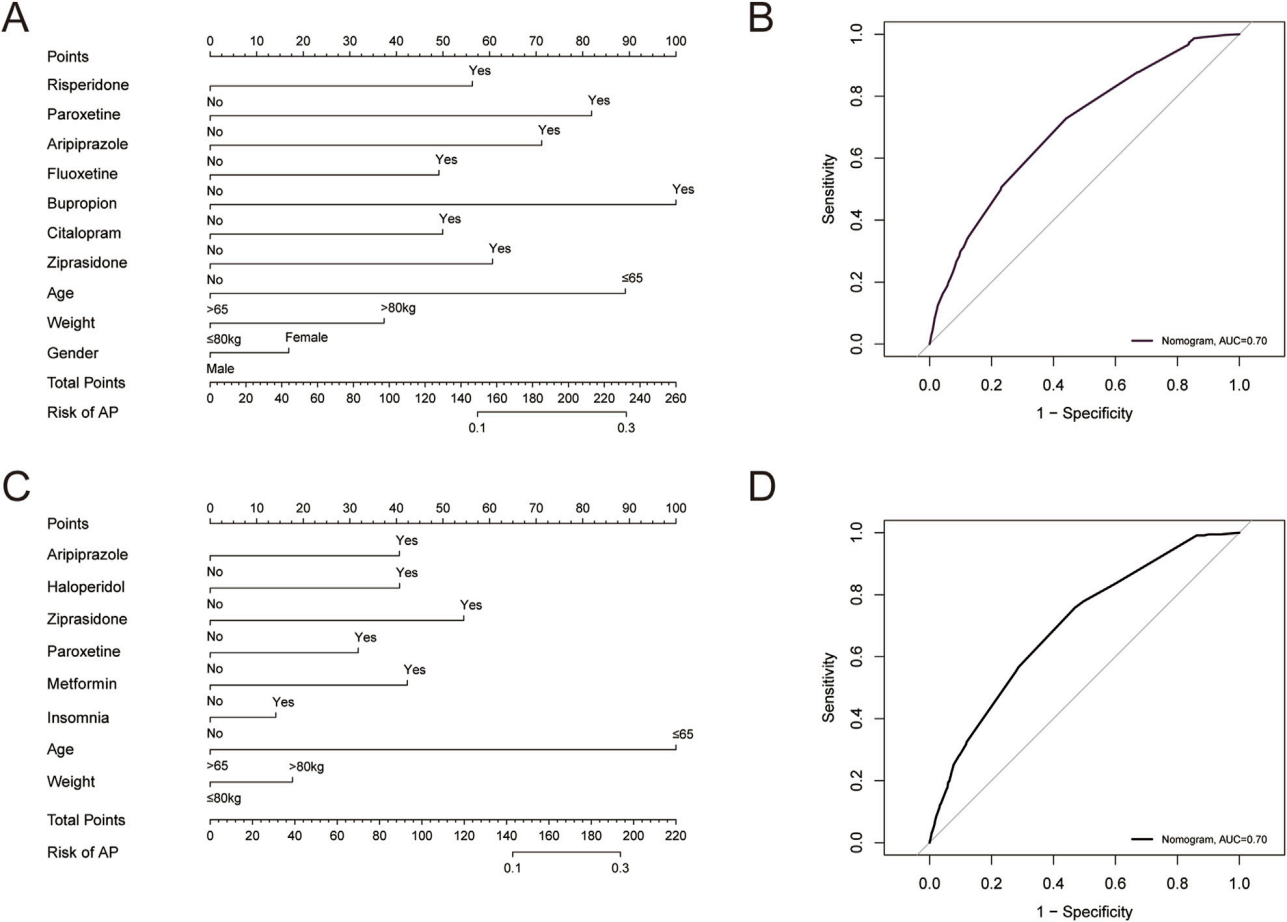
**(A)** A predicting nomogram of AP in patients receiving olanzapine. **(B)** A receiver operating characteristic (ROC) curve analysis of the predicting nomogram of AP in patients receiving olanzapine. **(C)** A predicting nomogram of AP in patients receiving quetiapine. **(D)** ROC curve analysis of the predicting nomogram of AP in patients receiving quetiapine.

To further examine risk factors that may influence the occurrence of AP, we performed univariate and multivariate logistic regression analyses (Table 5). In patients treated with olanzapine, age, gender, and weight can all independently affect the occurrence of AP. Female patients, patients aged <=65 years, and patients weighed >80 kg were more likely to experience AP. In patients receiving quetiapine, age and weight can independently affect the occurrence of AP. Patients aged <=65 years and weighed >80 kg were more likely to experience AP. Comorbidities and concurrent medications may also affect the occurrence of AP, therefore, we further investigated the impact of them. Combining olanzapine with risperidone, paroxetine, aripiprazole, fluoxetine, bupropion, citalopram, or ziprasidone could significantly increase the risk of developing AP (p < 0.05) (Table 5). We did not find comorbidities that might have impact on the risk of developing AP (Table 5). As shown in Figure 2A, we developed a nomogram model based on the age, sex, gender, and the seven concurrent medications. The AUC value of the model was 0.70, within an acceptable range (Figure 2B). For quetiapine, we observed that patients with insomnia might be at a higher risk of developing AP than those without it (p < 0.05) (Table 5). In terms of concurrent medications, the use of quetiapine in combination with aripiprazole, haloperidol, ziprasidone, paroxetine, metformin could significantly increase the risk of developing AP induced by quetiapine (p < 0.05) (Table 5). According to the results of logistic analysis, we further developed a nomogram model based on age, weight, insomnia, and the five concurrent medications (Figure 2C). The AUC value of the model was 0.70, indicating that the model had an effective forecasting ability (Figure 2D).

**TABLE 5:** Logistic regression analysis.

Drug	Subgroup		P (univariable)	OR (univariable)	P (multivariable)	OR (multivariable)
Olanzapine	Age	>65	Reference			
		≤65	<0.001	5.84 (3.49–10.75)	<0.001	4.26 (2.53–7.87)
	Gender	Male	Reference			
		Female	0.04	1.17 (1.01–1.36)	0.013	1.32 (1.06–1.64)
	Weight	≤80	Reference			
		>80	<0.001	2.21 (1.79–2.74)	<0.001	1.84 (1.47–2.30)
	Concurrent medications	Risperidone	0.003	2.25 (1.27–3.75)	0.001	2.50 (1.39–4.22)
		Paroxetine	<0.001	3.47 (2.02–5.67)	<0.001	3.79 (2.18–6.29)
		Aripiprazole	<0.001	3.17 (2.19–4.50)	<0.001	3.18 (2.17–4.57)
		Fluoxetine	<0.001	2.24 (1.39–3.46)	0.001	2.22 (1.36–3.47)
		Bupropion	<0.001	4.56 (2.53–7.81)	<0.001	5.08 (2.79–8.83)
		Citalopram	0.008	2.01 (1.16–3.30)	0.003	2.25 (1.28–3.73)
		Ziprasidone	0.003	2.74 (1.35–5.08)	0.004	2.68 (1.31–5.02)
Quetiapine	Age	>65	Reference			
		≤65	<0.001	18.61 (8.60–52.11)	<0.001	14.24 (6.56–39.95)
	Gender	Male	Reference			
		Female	0.004	0.78 (0.66–0.92)	0.058	0.85 (0.71–1.01)
	Weight	≤80	Reference			
		>80	<0.001	2.16 (1.82–2.58)	<0.001	1.60 (1.33–1.92)
	Comorbidities	Insomnia	0.008	1.40 (1.08–1.79)	0.004	1.45 (1.12–1.87)
	Concurrent medications	Aripiprazole	<0.001	3.18 (2.41–4.14)	<0.001	2.94 (2.22–3.86)
		Haloperidol	<0.001	3.02 (1.86–4.68)	<0.001	2.95 (1.80–4.60)
		Ziprasidone	<0.001	4.54 (3.16–6.38)	<0.001	4.25 (2.94–6.01)
		Paroxetine	0.025	1.99 (1.04–3.48)	0.007	2.33 (1.20–4.11)
		Metformin	0.003	2.75 (1.32–5.13)	0.001	3.08 (1.47–5.82)

### 3.5 Time to onset analysis

**FIGURE 3:**
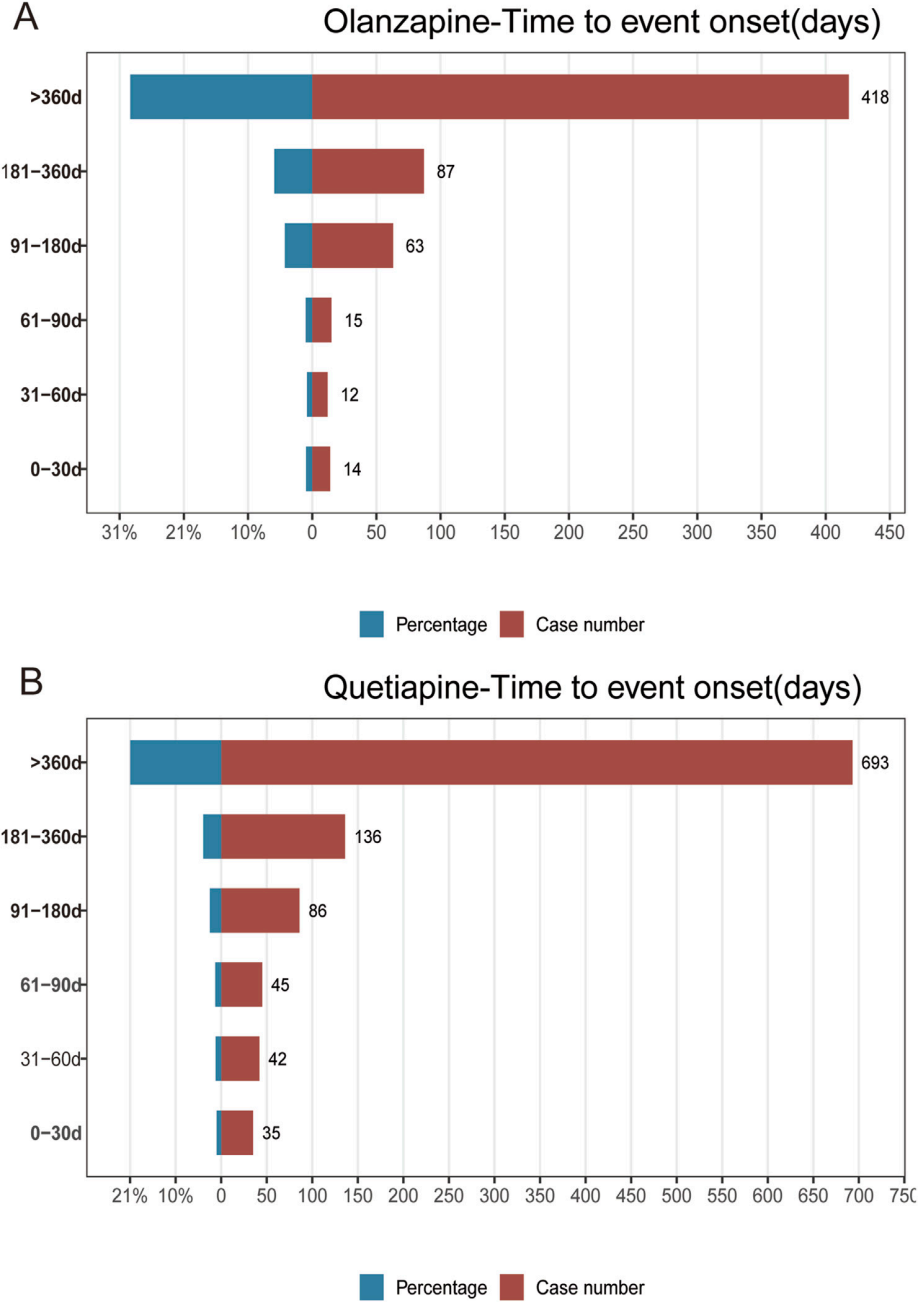
Results of time to event onset analyses of AP following the treatment of olanzapine **(A)**, and quetiapine **(B)**.

We further performed TTO analysis and WSP test to investigate the onset time of AP in associated with olanzapine and quetiapine, the results were shown in Figure 3 and Table 6. The median TTO and interquartile range (IQR) for olanzapine and quetiapine were 602 (269–1,369), and 574 (243–1,096) days. This highlighted the importance to be fully attentive to the signs of AP that may emerge after 602 days of treatment with olanzapine and 574 days of treatment with quetiapine. The results of WSP test of olanzapine and quetiapine indicated a random failure type, suggested that AP following the treatment of olanzapine and quetiapine continues to occur over time.

**TABLE 6:** Results of TTO analysis and WSP test.

Drug	TTO analysis		Scale parameter		Shape parameter		Type
	Median (IQR)	Min-max	α	95%CI	β	95%CI	
Olanzapine	602 (269–1,369)	1–7,256	915.67	840.34–991.01	1.02	0.96–1.08	Random failure type
Quetiapine	574 (243–1,096)	1–5,113	799.47	749.07–849.87	1.01	0.97–1.06	Random failure type

TTO: time-to-onset, WSP: weibull distribution, IQR: interquartile range.

### 3.6 Identification of genes related to AP

**FIGURE 4:**
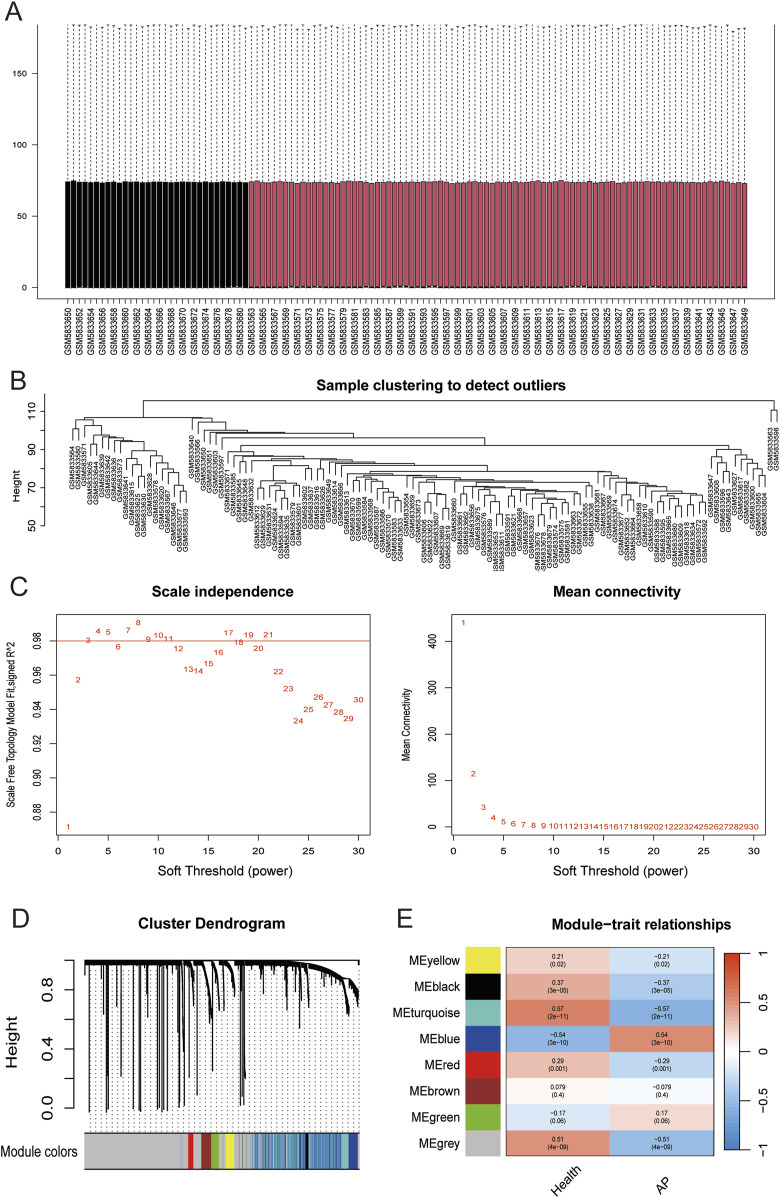
**(A)** Box plots of raw data normalized between samples. **(B)** Sample clustering to detect outliers. **(C)** Choosing the best soft-threshold power. **(D)** By aggregating genes with strong correlations in the same module, different modules were obtained and are displayed in different colors. **(E)** Eight modules revealed by the WGCNA. WGCNA, weighted gene co-expression network analysis.

The original AP dataset GSE194331 was collected from the GEO database and included peripheral blood gene expression data of 87 AP patients and 32 healthy patients. Normalization of GSE194331 was performed, and the results were visualized in Figure 4A. We employed WGCNA to identify the most relevant modules in AP. The clustering tree shows that the samples have excellent clustering properties, with no obvious outlier samples detected (Figure 4B). To ensure a scale-free distribution of the connections between genes in the network, we selected a soft threshold power of β = 8 (scale-free R^2^ = 0.991) and estimated the average connectivity and scale-free fit index (Figure 4C). Using the dynamic cutting method, we created 8 co-expression modules of different colors (Figure 4D). Subsequently, we plotted the heatmap between these module eigengenes (MEs) and clinical traits (Figure 4E). The turquoise module showed the most significant negative correlation with AP (r = −0.57, P = 2e-11), whereas the bule module displayed the strongest positive correlation with AP (r = 0.54, P = 3e-10). Combine genes in turquoise module and bule module, a total of 1,085 genes were identified to be key genes closely associated with AP.

### 3.7 Retrieval of potential drug targets

**FIGURE 5:**
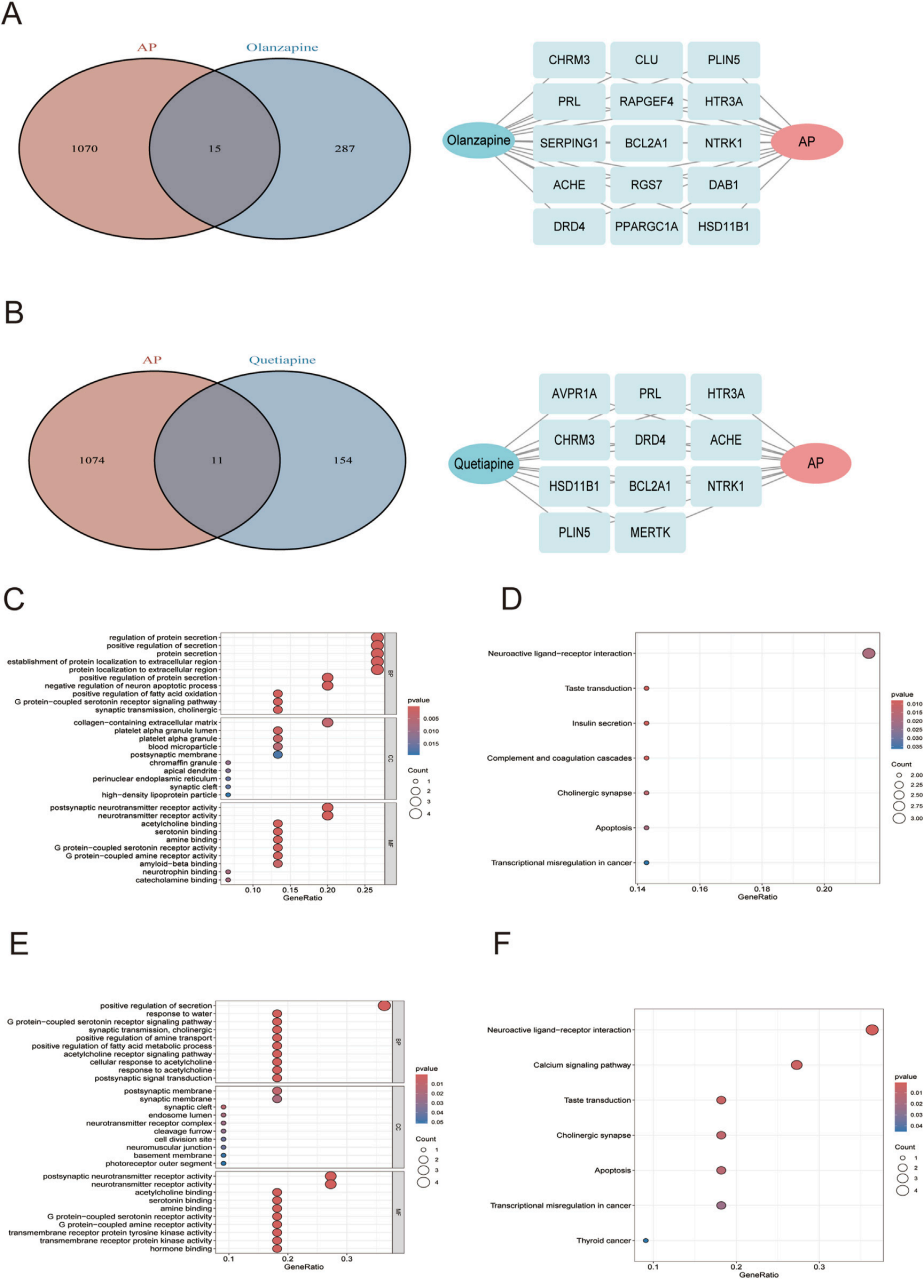
**(A)** Left: venn diagrams of olanzapine candidate targets against AP; right: the 15 overlapping genes. **(B)** Left: venn diagrams of quetiapine candidate targets against AP; right: the 11 overlapping genes. The GO **(C)** and KEGG **(D)** plots of the 15 olanzapine candidate targets against AP. The GO **(E)** and KEGG **(F)** plots of the 11 quetiapine candidate targets against AP.

Synthesizing PubChem database, Comparative Toxicogenomics Database, Swiss Target Prediction database, BindingDB, TargetNet, UniProt and STRING, a total of 302 genes were identified to be the potential targets of olanzapine (Supplementary Table S4), 165 genes were identified to be the potential targets of quetiapine (Supplementary Table S5). By intersecting these gene targets with 1,085 key genes closely related to AP, 15 overlapping genes were selected as candidate targets for olanzapine against AP (Figure 5A), and 11 overlapping genes were selected as candidate targets for quetiapine against AP (Figure 5B). For olanzapine, GO enrichment analysis (Figure 5C) showed several biological processes that were strongly linked to the 15 candidate targets against AP, which included regulation of protein secretion, positive regulation of secretion, establishment of protein localization to extracellular region, protein localization to extracellular region, and so on. Cellular component analysis revealed significant enrichment of collagen-containing extracellular matrix. Molecular function analysis shows that postsynaptic neurotransmitter receptor activity and neurotransmitter receptor activity were significantly enriched, highlighting the role of 15 candidate targets against AP in regulating neurotransmitter receptor activity. KEGG enrichment analysis further revealed significant enrichment of neuroactive ligand-receptor interaction (Figure 5D). For quetiapine, biological process analysis revealed significant enrichment of positive regulation of secretion, G protein−coupled serotonin receptor signaling pathway, positive regulation of fatty acid metabolic process, acetylcholine receptor signaling pathway, and so on. Cellular component analysis showed significant enrichment of the postsynaptic membrane. Molecular functional analysis revealed significant enrichment of neurotransmitter receptor activity, serotonin binding, G protein−coupled serotonin receptor activity and so on (Figure 5E). KEGG (Figure 5F) analysis further suggested significant enrichment of neuroactive ligand-receptor interaction, and revealed the enrichment of calcium signaling pathway, taste transduction, cholinergic synapse, and so on.

### 3.8 Construction of PPI networks and functional analysis

**FIGURE 6:**
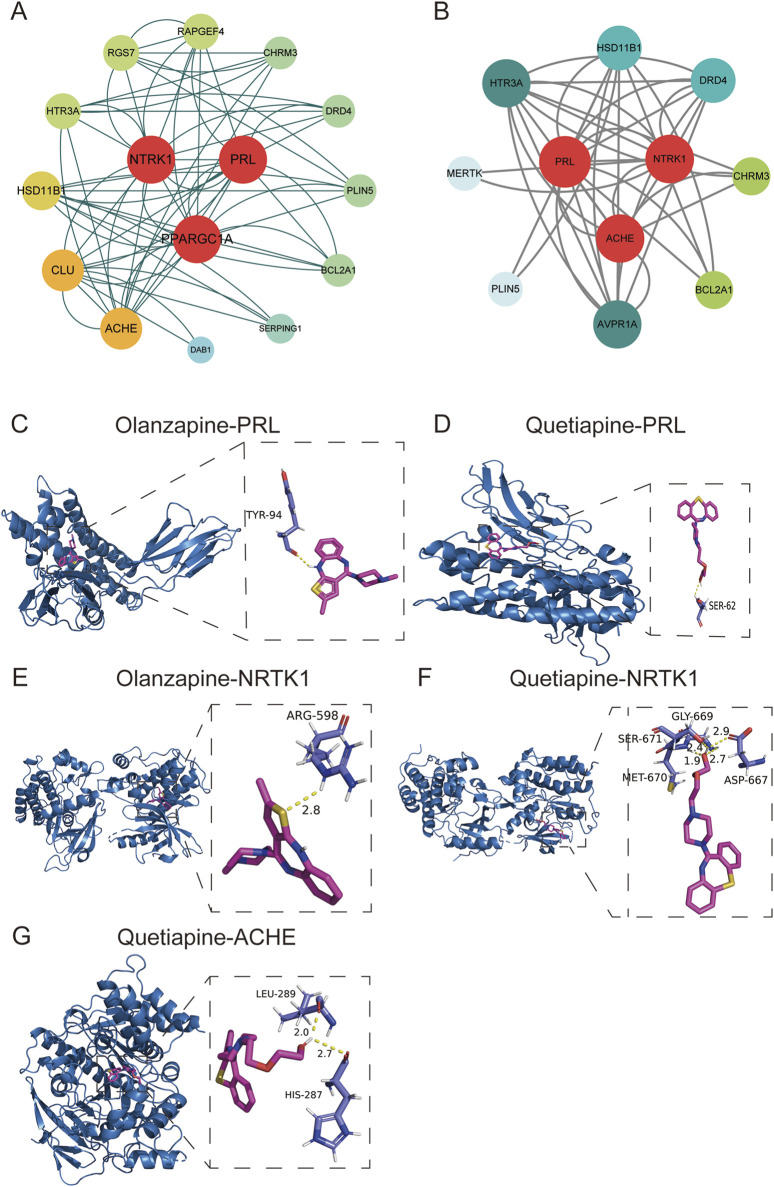
PPI networks for olanzapine **(A)** and quetiapine **(B)**. **(C)** Docking plots of olanzapine with PRL. **(D)** Docking plots of quetiapine with PRL. **(E)** Docking plots of olanzapine with NTRK1. **(F)** Docking plots of quetiapine with NTRK1. **(G)** Docking plots of quetiapine with ACHE.

PPI networks were constructed for olanzapine and quetiapine using candidate gene targets against AP, respectively. The redder and larger notes indicated genes with higher DC value. Nodes with higher DC values were considered more important in the network and the top 3 genes with highest DC values were identified as hub genes. For olanzapine, NTRK1 (neurotrophic tropomyosin kinase receptor 1), PPARGC1A (coactivator-1alpha), and PRL (prolactin) were identified as potential hub genes (Figure 6A). For quetiapine, PRL, ACHE (acetylcholinesterase), and NTRK1 were considered as potential hub genes (Figure 6B). To further verify whether olanzapine and quetiapine can bind to their hub genes and thus mediate the development of AP, we conducted molecular docking analyses.

### 3.9 Validation of molecular docking between olanzapine, quetiapine and hub genes

The protein model of PRL (3N06), NTRK1 (4F0I), PPARGC1A (8BF1), and ACHE (6O69) were downloaded from the PDB database. The interactions between olanzapine, quetiapine and proteins were analyzed by Auto Dock Vina 1.2.0 and visualized with PyMOL (2.5.7). The binding affinity between olanzapine and PRL, NTRK1 were −7.6 and −8.9 kcal/mol, respectively, indicating that there was a good binding ability between olanzapine and the two proteins. As shown in Figure 6C, olanzapine bond to TYR-94 within PRL, forming one hydrogen bond. Within NTRK1 protein, olanzapine bond to ARG-598, forming one hydrogen bond (Figure 6E). However, the minimum binding free energy of olanzapine and PPARGC1A was 2.9 kcal/mol, suggesting that the combination between them was unstable. The docking scores of quetiapine and PRL, NTRK1, and ACHE were −6.5, −8.9, and −7.9 kcal/mol, respectively, suggesting that the three proteins had good binding activities with quetiapine. As shown in Figure 6D, quetiapine bond to SER-62 within PRL protein, forming one hydrogen bond. Within NTRK1 protein, quetiapine bond to four residues (SER-671, MET-670, GLY-669, and ASP-667), forming four hydrogen bonds (Figure 6F). Within ACHE protein, quetiapine bond to two residues (LEU 289 and HIS 287), forming two hydrogen bonds (Figure 6G).

## 4 Discussion

### 4.1 Analysis of pharmacovigilance signals

AP induced by antipsychotics was overall infrequent but could be potentially life-threatening. This study compiled information on the risk of AP associated with antipsychotics through the disproportionality analysis of spontaneous reports of FAERS database. Among a total of 63 antipsychotics active ingredients, ROR, PRR, BCPNN, and EBGM results for olanzapine, quetiapine, and fluphenazine were observed to be statistically significant, indicating a potential association between AP and these drugs. However, in the FAERS database, only 7 reports of fluphenazine were found to be related to AP. Considering the small sample size, we mainly focused on AP associated with olanzapine and quetiapine in the further analyses.

AP caused by quetiapine had a higher risk intensity than that of olanzapine according to ROR, PRR, BCPNN, and EBGM methods, which may indicate that patients treated with quetiapine are more likely to develop AP than patients receiving olanzapine. Several case reports reported the occurrence of AP shortly after the initiation of quetiapine. For example, Liou et al. reported a patients developed acute pancreatitis after quetiapine administration (Liou et al., 2014). The mechanism of AP induced by quetiapine is complex and remains incompletely understood. However, existing studies have provided clues from multiple perspectives, as detailed below: (i) the use of quetiapine may lead to elevated triglyceride levels, and chylomicrons may obstruct capillaries, thereby causing AP (Liou et al., 2014); (ii) excessive triglycerides are hydrolyzed by pancreatic lipase, leading to the accumulation of free fatty acids, which can damage pancreatic capillaries and cause local tissue ischemia, ultimately resulting in AP (Liou et al., 2014); (iii) promoted by inflammatory reactions: excess free fatty acids in triglycerides can cause further cytotoxic damage, releasing inflammatory factors and exacerbating the condition of AP. To further investigate the clinical characteristics of the occurrence of AP in patients receiving quetiapine, we conducted subgroups analysis based on age, gender, and weight. According to age classification criteria of the World Health Organization, individuals aged 0–65 years were identified as juvenile and young, individuals aged >65 years were identified as middle-aged and elderly. To further investigate the clinical characteristics of the occurrence of AP in patients receiving quetiapine, we conducted subgroups analysis based on age, gender, and weight. According to age classification criteria of the World Health Organization, individuals aged 0–65 years were identified as juvenile and young, individuals aged >65 years were identified as middle-aged and elderly (Sut et al., 2023; Ergin et al., 2021). With the improvement of people’s health conditions, the incidence of AP in middle aged and elderly population has shown to be gradually increased (Quero et al., 2020). It is worth noting that approximately one-third of patients diagnosed with acute pancreatitis in the emergency department are aged ≥65 years old (Gloor et al., 2002). Therefore, we set 65 years as the cut-off value for age to preliminarily explore the occurrence of AP induced by olanzapine and quetiapine. In terms of weight, due to the lacking of height information, we set a cut off value to 80 kg based on previous researches (Deng et al., 2024).

In the further investigation of clinical characteristics of the occurrence of AP in patients receiving quetiapine, we found the results of ROR, PRR, BCPNN, and EBGM methods were statistically significant in age<=65, male, female, weight<=80, and weight >80 kg subgroups, indicating the robustness of our results. Combined with the results of univariate and multivariate logistic regression, weight >80 kg and age <=65 were observed as risk factors of the occurrence of AP in patients receiving quetiapine. A systematic review conducted by Lucia et al. showed that quetiapine increased body weight >=7% from baseline (Alonso-Pedrero et al., 2019). Overweight and obese people often have higher levels of triglycerides (Howard et al., 2003). Excess triglycerides hydrolyzed by pancreatic lipase can lead to accumulation of free fatty acids, resulting in pancreatic capillary damage and local tissue ischemia, and thus causing AP (Lin et al., 2022). A possible reason for why age <=65 was the risk factor may be that compared to the older age group, people in this age group had higher proportion of quetiapine use (Taipale et al., 2024; Al-Qaaneh et al., 2023).

In our study, all the for algorithms showed that there were statistically significant results for olanzapine. Consistently with our study, Sivapriya found AP can occur in patients receiving olanzapine (Vaidyanathan et al., 2019). There are several reasonable hypotheses regarding the mechanism by AP caused by olanzapine: (i) metabolic adverse reactions, especially hyperlipidemia, hyperglycemia, and weight gain, may indirectly induce AP (Weston-Green et al., 2012; Sussman, 2003; Albaugh et al., 2012); (ii) Genetic variations, especially polymorphisms in the CYP2D6, CYP1A2, UGT1A4, and FMO3 coding genes, may affect the metabolism of olanzapine (Ustohal et al., 2016; Lett et al., 2012). According to the four pharmacovigilance methods, the signal intensity in the subgroup of age <=65, male, female, weight <=80 kg, and weight >80 kg, were all observed to be statistically significant. In the further investigation, we found age <=65, female, and weight >80 kg were the risk factors of the occurrence of AP in patients receiving olanzapine. Consistent with our study, Matthew et al. also found patients with AP were tend to be younger. The specific mechanism still needs to be further explored. CYP1A2 participated in the metabolism of olanzapine (Rodenburg et al., 2012). Huang et al. found the expression level of CYP1A2 was lower in females than males, leading to higher systemic exposure of olanzapine in females, this may explain why female treated with olanzapine are more likely to develop AP. Obesity causes paracrine effects on cell function in the pancreas (Lilly et al., 2023). Paracrine effects can improve the secretion of adipokines and pro-inflammatory cytokines, causing accumulation of lipid droplets and fat cells, creating a disease-promoting metabolic environment, and thus leading to AP (Petrov and Taylor, 2022; Shulman, 2014).

This study also found that combining olanzapine with risperidone, paroxetine, aripiprazole, fluoxetine, bupropion, citalopram, ziprasidone could significantly increase the risk of developing AP (p < 0.05). It has been suggested that long-term use of risperidone may trigger the elevated level of triglycerides and cholesterol, which may help trigger the development of AP (Deng et al., 2022; Kawabe and Ueno, 2014). A case reported by Vucicevic found that simultaneously administrate paroxetine and olanzapine might develop AP, which was consistent with the results of our study (Vucicevic et al., 2007). Matthew et al. found aripiprazole exposure may be associated with the development of AP (Silva et al., 2016). The association between fluoxetine and AP was controversial. Several case reports have reported the development of AP after taking fluoxetine (Chahed et al., 2024). However, a case-control study showed that the use of fluoxetine was not significantly associated with the occurrence of AP (Ljung et al., 2012). The results of our study suggested that combining olanzapine with fluoxetine could significantly increase the risk of developing AP. Bucklin et al. found that bupropion may associated with AP, which was consistent with our study (Bucklin et al., 2013). The study of Matthew et al. suggested the application of ziprasidone was associated with AP (Silva et al., 2016). Up to now, few articles have explored whether or not the combination of those drugs with olanzapine may increase the risk of AP. Our results provide some references for the clinical application of these drugs. However, the underlying mechanism still needs to be further explored. For quetiapine, we observed that patients with insomnia might be at a higher risk of developing AP than those without it. Previous studies have found that insomnia could lead to increases in inflammation (Irwin et al., 2016). It is known that AP is a type of inflammatory disease, the association between insomnia and inflammation may help to explain the higher risk of developing AP when insomnia was as comorbidity. In terms of concurrent medications, the use of quetiapine in combination with aripiprazole, haloperidol, ziprasidone, paroxetine, or metformin could significantly increase the risk of developing AP induced by quetiapine. These results provide some reference for clinicians to choose the concurrent medications of olanzapine and quetiapine and help assess the risk of AP in patients with insomnia who receive olanzapine or quetiapine.

According to the results of our study, AP following olanzapine and quetiapine use occurred at a median of 602 and 574 days, respectively. This period is crucial for detecting and managing AP in patients receiving olanzapine and quetiapine, thereby optimizing safety of patient and treatment outcomes.

The findings of our study have important implications for AP prevention of antipsychotics. Patients taking olanzapine and quetiapine should be aware of the potential risk of AP and should report any AP-related symptoms to their healthcare provider immediately. Doctors should monitor patients taking olanzapine and quetiapine for signs and symptoms of AP and withdraw the medication if AP is suspected.

### 4.2 Analysis of potential toxicological mechanisms of AP induced by olanzapine and quetiapine

We further explored toxicological mechanism of AP induced by olanzapine and quetiapine. Currently, the diagnosis of AP is mainly based on laboratory data, clinical findings, and imaging (Phillip et al., 2014). Because of the relative inaccessibility of pancreatic tissue and the rapid course of AP, obtaining blood samples rather than pancreatic tissue seems to be a practical and cost-effective way to establish early diagnosis (Liu and Dai, 2023). As a type of inflammatory disease, AP begins with the occurrence of local inflammation of the pancreas and soon progresses into a generalized inflammatory response (Li et al., 2021; Bhatia et al., 2000). In the initial stage of AP, the immune system in the blood has been activated (Liu et al., 2022). With the progress of high-throughput technologies, blood transcriptomic profiling has been proven to be a powerful way in elucidating the pathogenesis and course of infectious diseases, autoimmune diseases, and cancer (Bennett et al., 2003; Pankla et al., 2009). As the only publicly available human peripheral blood transcriptome dataset for AP, GSE194331 has been widely used for the investigation of reliable biomarkers in the diagnosis of AP and has been validated to have good diagnostic value for AP. For example, by exploring GSE194331, Zhang et al. found that S100A6, S100A9, and S100A12 were good predictors of severe AP, which have been validated using blood samples from AP patients (Zhang et al., 2022). Therefore, we believe that GSE194331 has good value in characterizing the pathophysiological characteristics of AP. By exploring GSE194331, we can better explore the possible mechanisms by which olanzapine and quetiapine induce AP. A total of 15 genes were selected as candidate targets of AP induced by olanzapine, 11 genes were identified as quetiapine candidate targets of AP induced by quetiapine. PPI networks revealed that NTRK1, PPARGC1A, and PRL were hub genes of olanzapine-induced AP, while PRL, ACHE, and NTRK1 were potential hub genes of quetiapine-induced AP. The results of molecular docking showed that there was a good binding ability between olanzapine and PRL and NTRK1, while PRL, ACHE, and NTRK1 also had good binding activities with quetiapine.

The PRL gene is composed of four introns and five exons, and is located on chromosome 6, encoding prolactin (Vera-Lastra et al., 2002). Elevated prolactin levels are a common side effect in patients taking olanzapine and quetiapine (Zhang et al., 2018). Previous study has demonstrated that PRL level may elevate proinflammatory immune responses, and thus playing an important role in immune dysfunctions (Cejkova et al., 2009). PRL can activate the JAK/STAT signaling pathway by binding to its receptor PRLR, aggravate the inflammatory response in the pancreas (Cejkova et al., 2009; Lesina et al., 2014). In this study, we tested the affinity of olanzapine and quetiapine with PRL using docking technology, and the two drugs were found to bind closely to PRL. Although PRL itself can promote the development of AP through inflammatory response, whether the complex produced by PRL combined with olanzapine or quetiapine can promote the development of AP needs to be further explored.

The NTRK1 gene encodes neurotrophic tropomyosin kinase receptor 1, which plays an important role in the regulation of cell survival and differentiation (Rogers et al., 2022). This receptor is part of the neurotrophin signaling pathway, which is critical for neuronal growth, maintenance, and plasticity. Dysregulation of NTRK1 has been implicated in various pathological conditions, including inflammatory responses and tissue damage. In this study, molecular docking revealed a strong binding affinity between olanzapine, quetiapine, and NTRK1, suggesting that these drugs may modulate NTRK1-mediated signaling pathways. Specifically, olanzapine formed a hydrogen bond with ARG-598 within NTRK1, while quetiapine interacted with multiple residues (SER-671, MET-670, GLY-669, and ASP-667), forming four hydrogen bonds. These interactions could potentially disrupt NTRK1’s normal function, contributing to the development of acute pancreatitis (AP) by altering cellular survival and inflammatory responses in pancreatic tissues. However, further experimental validation is needed to elucidate the exact mechanisms by which NTRK1 influences AP pathogenesis when bound to these antipsychotics.

The human ACHE gene, which is seven kilobases in size, is located at the q22 of the long arm of chromosome 7 (7q22). It is comprised of six exons and five intronic regions (Richbart et al., 2021). This ACHE gene encodes acetylcholinesterase, an enzyme responsible for the breakdown of acetylcholine, a key neurotransmitter in the cholinergic system. Acetylcholinesterase plays a crucial role in terminating synaptic transmission and maintaining proper neuromuscular function. Dysregulation of ACHE activity has been linked to inflammatory processes and oxidative stress, which are central to the pathogenesis of AP. In this study, quetiapine exhibited a strong binding affinity with ACHE, forming hydrogen bonds with residues LEU 289 and HIS 287. This interaction could potentially inhibit ACHE activity, leading to an accumulation of acetylcholine and subsequent overstimulation of cholinergic pathways. Such overstimulation may exacerbate pancreatic inflammation and acinar cell damage, thereby promoting AP (Mitchell et al., 1994). The involvement of ACHE in quetiapine-induced AP highlights the complex interplay between neurotransmitter systems and pancreatic pathology. Further research is warranted to explore whether ACHE inhibition by quetiapine directly contributes to AP or serves as a secondary effect of broader metabolic disturbances.

### 4.3 Limitations

Our study has certain limitations. First, as a spontaneous reporting system, reporting and information bias exist in the FAERS database. Moreover, FAERS often contains missing information and lacks data on population exposure, precluding us from calculating incidence rates of AP (Bate and Evans, 2009). Third, causality of the drug-induced AP could not be confirmed by the reports. Forth, in network toxicology, the interactions between olanzapine, quetiapine, and PRL have not been validated in any vivo and *in vitro* experiments. Animal experiments and clinical trials are needed to confirm the findings. Fifth, because the FAERS database did not record height information for patients, we were unable to explore the potential impact of BMI. Finally, the level of TCA in patients receiving olanzapine or quetiapine may help to explain the potential mechanisms of developing AP, however, because the FAERS database did not record the relevant information, we were unable to measure the level of TCA during AP attack, which is also a limitation of our study.

## Data Availability

Publicly available datasets were analyzed in this study. This data can be found here: https://www.fda.gov/drugs/fdas-adverse-event-reporting-system-faers/fda-adverse-event-reporting-system-faers-public-dashboard; and here: https://www.ncbi.nlm.nih.gov/geo/query/acc.cgi?acc=GSE194331.
