# Supplementary material for: An in-depth exploration of the association between olanzapine, quetiapine and acute pancreatitis based on real-world datasets and network toxicology analysis
Source: Front Pharmacol. 2025 May 2;16:1529416. doi: 10.3389/fphar.2025.1529416 (PMC12082570; doi:10.3389/fphar.2025.1529416)
Supplement: Supplementary file 3 [file Table1.docx]

Supplementary Table 1. Antipsychotic drugs

| Chlorpromazine |
| --- |
| Levomepromazine |
| Promazine |
| Acepromazine |
| Triflupromazine |
| Cyamemazine |
| Chlorproethazine |
| Dixyrazine |
| Fluphenazine |
| Perphenazine |
| Prochlorperazine |
| Thiopropazate |
| Trifluoperazine |
| Acetophenazine |
| Thioproperazine |
| Periciazine |
| Thioridazine |
| Mesoridazine |
| Pipotiazine |
| Haloperidol |
| Trifluperidol |
| Melperone |
| Moperone |
| Pipamperone |
| Bromperidol |
| Benperidol |
| Droperidol |
| Fluanisone |
| Oxypertine |
| Molindone |
| Sertindole |
| Ziprasidone |
| Lurasidone |
| Flupentixol |
| Clopenthixol |
| Chlorprothixene |
| Tiotixene |
| Zuclopenthixol |
| Fluspirilene |
| Pimozide |
| Penfluridol |
| Loxapine |
| Clozapine |
| Olanzapine |
| Quetiapine |
| Asenapine |
| Clotiapine |
| Levosulpiride |
| Sultopride |
| Tiapride |
| Remoxipride |
| Amisulpride |
| Veralipride |
| Lithium |
| Prothipendyl |
| Risperidone |
| Mosapramine |
| Zotepine |
| Aripiprazole |
| Paliperidone |
| Iloperidone |
| Cariprazine |
| Levosulpiride |
