# Supplementary material for: An in-depth exploration of the association between olanzapine, quetiapine and acute pancreatitis based on real-world datasets and network toxicology analysis
Source: Front Pharmacol. 2025 May 2;16:1529416. doi: 10.3389/fphar.2025.1529416 (PMC12082570; doi:10.3389/fphar.2025.1529416)
Supplement: Supplementary file 4 [file Table2.docx]

Supplementary Table 2. PTs contained in the narrow-scope search of “Acute pancreatitis (SMQ)” (MedDRA code: 20000022)

| Cullen's Sign, |
| --- |
| Grey Turner's Sign, |
| Haemorrhagic Necrotic Pancreatitis, |
| Hereditary Pancreatitis, |
| Idiopathic Pancreatitis, |
| Immune-Mediated Pancreatitis, |
| Ischaemic Pancreatitis, |
| Oedematous Pancreatitis, |
| Pancreatic Abscess, |
| Pancreatic Cyst Drainage, |
| Pancreatic Haemorrhage, |
| Pancreatic Phlegmon, |
| Pancreatic Pseudoaneurysm, |
| Pancreatic Pseudocyst, |
| Pancreatic Pseudocyst Drainage, |
| Pancreatic Pseudocyst Haemorrhage, |
| Pancreatic Pseudocyst Rupture, |
| Pancreatitis, |
| Pancreatitis Acute, |
| Pancreatitis Haemorrhagic, |
| Pancreatitis Necrotising, |
| Pancreatitis Relapsing, |
| Pancreatorenal Syndrome, |
| Subacute Pancreatitis, |
| Walled-Off Pancreatic Necrosis |
